# Sturge-Weber syndrome with cemento-ossifying fibroma in the maxilla and giant odontoma in the mandible: A case report

**DOI:** 10.1016/j.heliyon.2024.e29445

**Published:** 2024-04-15

**Authors:** Aki Sato, Hisako Furusho, Tatsushi Matsumura, Makoto Nakano, Koichi Sawaki, Yohsuke Yoshioka, Sho Akashi, Mutsumi Miyauchi, Nobuyoshi Mizukawa, Seiji Iida

**Affiliations:** a Department of Dentistry and Oral Surgery, Hiroshima City Hiroshima Citizens Hospital, Hiroshima, Japan; b Department of Pathology, Hiroshima City Hiroshima Citizens Hospital, Hiroshima, Japan; c Department of Oral and Maxillofacial Pathobiology, Hiroshima University Graduate School of Biomedical and Health Sciences, Hiroshima, Japan; d Department of Oral and Maxillofacial Surgery, Wakayama Medical University, Wakayama, Japan; e Department of Dentistry, Kurashiki Central Hospital, Okayama, Japan; f Department of Oral and Maxillofacial Reconstructive Surgery, Okayama University Hospital, Okayama, Japan; g Department of Oral and Maxillofacial Reconstructive Surgery, Okayama University Graduate School of Medicine, Dentistry and Pharmaceutical Sciences, Okayama, Japan

## Abstract

Sturge-Weber syndrome (SWS) is a neurocutaneous syndrome with vascular lesions of the cerebral meninges, port wine spots on the face, and glaucoma of the eyes; it is a congenital, non-genetic disease whose etiology and mechanisms are unknown. In this report, we describe a rare case of SWS with unilateral large odontogenic tumors in the maxilla and mandible. The histopathological diagnosis of the maxillary bone lesion on biopsy was juvenile psammomatoid ossifying fibroma, which is considered a type of ossifying fibroma of craniofacial bone origin. However, the final pathological diagnosis of the excision was cemento-ossifying fibroma derived from periodontal ligament cells, and we discuss the histopathology in detail. In addition, the mandibular lesion was one of the largest odontomas reported to date. Furthermore, in this case, we suggest the possibility that the maxillary and mandibular bone lesions are not separate lesions, but a series of lesions related to SWS.

## 1 Introduction

Sturge-Weber syndrome (SWS) was first described by Sturge in 1879 [1], and Weber reported calcifications on skull radiographs in 1922 [2]. It is characterized by unilateral port wine spots (capillary malformations) on the face, leptomeningeal angioma, and ocular manifestations (eyelid and conjunctival abnormalities, glaucoma, choroidal hemangioma) occurring ipsilaterally on the face along with intractable epilepsy, mental retardation, and contralateral hemiplegia, which are clinically problematic. There is no sex difference in the occurrence, and the incidence is reported to be approximately 1 in 20,000–50,000 persons [3,4]. Although the exact cause of the disease is unknown, it is suggested to be due to venous insufficiency associated with retraction of the primitive venous plexus, which normally occurs at 5–8 weeks of gestation, resulting in vascular lesions in the cerebral meninges, facial skin, and eyes due to impaired venous perfusion [5,6]. Soft tissue overgrowth, osteodeformity, and osteohypertrophy have already been reported in many cases as oral features of SWS [[7], [8], [9]] but there are few reports involving jawbone tumors, and it is necessary to accumulate clinical case reports on the oral phenotype of SWS.

In this case, we describe a rare case of SWS with a cemento-ossifying fibroma (COF) in the maxilla and a giant odontoma in the mandible. We also discuss the relationship between maxillary and mandibular bony lesions and SWS, along with a review of previous literature.

## 2 Case report

**Fig. 1:**
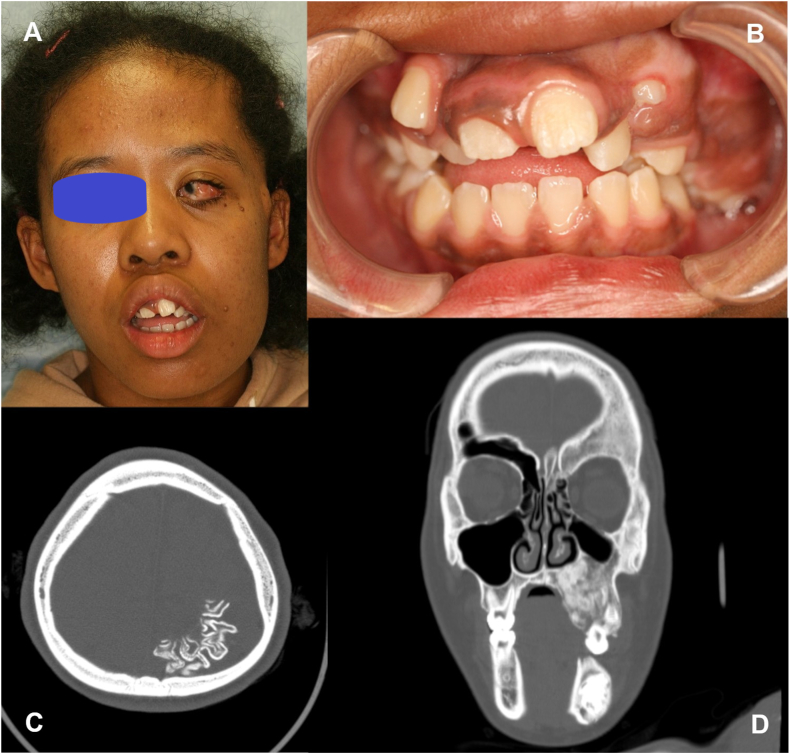
Clinical initial findings. A: Facial photo without port wine stains on the face, note a neoplastic lesion in the left eye. B: Intraoral view showing irregular dentition and the thickened and pigmented gingiva. C: Intracranial computed tomography image of the calcification of the left cerebral surface D: Coronal computed tomography image showing the hypoplastic cavity of the frontal and maxillary sinuses. There is a thickening of the left frontal bone and highly attenuating shadows with surrounding lucent bands are observed in the left maxilla and mandible.

**Fig. 2:**
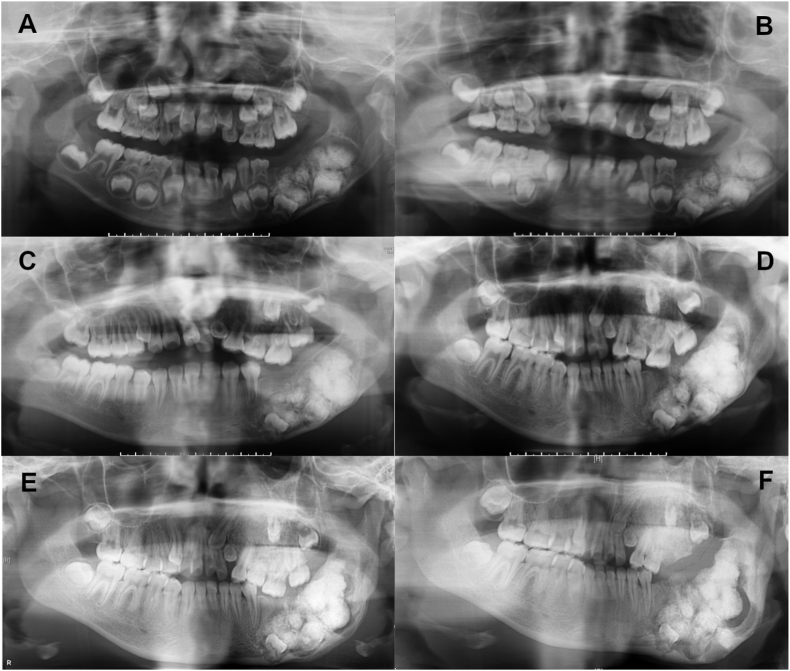
Successive findings in panoramic radiography A (at age 6): A hard tissue lesion in the left mandible was observed, and the left lower second deciduous molar was missing. From B (at age 7), C (at age 11), to D (at age 13): The hard tissues were calcified, and the boundary with the surrounding alveolar bone became indistinct. From E (age 14) to F (age 16): The boundary with the surrounding area gradually became more distinct. The lesion was believed to have originated from the left maxillary bone between B (at age 7) and C (at age 11).

**Fig. 3:**
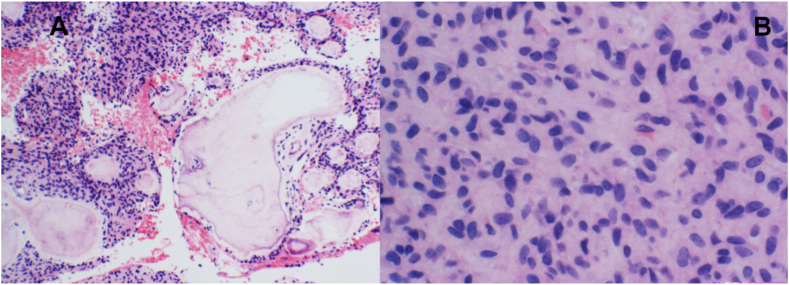
Histopathological images at the biopsy A,B: Tumor consists of dense spindle-shaped cell proliferation with cementum-like spherical hard tissues of various sizes (A, arrow, A, H&E; × 100). No cellular atypia was observed in the spindle-shaped cells (B, H&E; × 200).

A 14-year-old girl was noted to have an abnormality in her left mandible at the age of 6 years by a dentist, but was asymptomatic and was followed up. However, she complained of pain in the left mandible and was referred to our department. She had been diagnosed with Sturge-Weber syndrome at the age of 5 years and had epilepsy, mental retardation, cerebral palsy, right-sided upper and lower limb paralysis, left-sided exophthalmic angle cartilage nevus, and a left eye conjunctival choristoma. The patient had no relevant family history and was enrolled at an institution for children with disabilities. At the time of initial examination, port wine spots were not observed on the face; however, a choristoma was observed in the left eye. The facial appearance was slightly asymmetrical, with a bone-like hard bulge on the left mandibular body (Fig. 1A). Intraoral examination revealed dentition irregularities and thickened and pigmented gingiva (Fig. 1B). Computed tomography (CT) revealed calcification of the cerebral surface on the left side (Fig. 1C) and hypoplasia of the frontal and maxillary sinuses. There was also thickening of the left frontal bone and highly attenuating shadows with surrounding lucent bands were observed in the left maxilla and mandible (Fig. 1D). Panoramic radiographs showed a lesion with hard tissue in the left mandible by the age of 6 years, and the left lower second deciduous molar was missing (Fig. 2A). The hard tissues subsequently grew, and the boundary between the lesion and the surrounding alveolar bone initially became indistinct (Fig. 2BCE and D) but became more distinct later (Fig. 2E and F). The lesion in the left maxilla occurred in the tooth-bearing area of the jawbone between the ages of 7 and 11 years (Fig. 2B and C). In the same area, there was a frosted radiopaque image showing the embedded tooth of the left upper second premolar being pushed up as the lesion grew (Fig. 2CD and E). Biopsies were performed twice, and the maxillary lesion was diagnosed as a juvenile psammomatoid ossifying fibroma (JPOF; Fig. 3A and B). The mandibular lesion was pathologically diagnosed as complex odontoma. The patient was followed up for a period of growth, but the maxillary tumor had grown, and the lesion in the mandibular tumor had become frequently infected; therefore, maxillary tumor reduction and mandibular tumor excision were planned under general anesthesia.

**Fig. 4:**
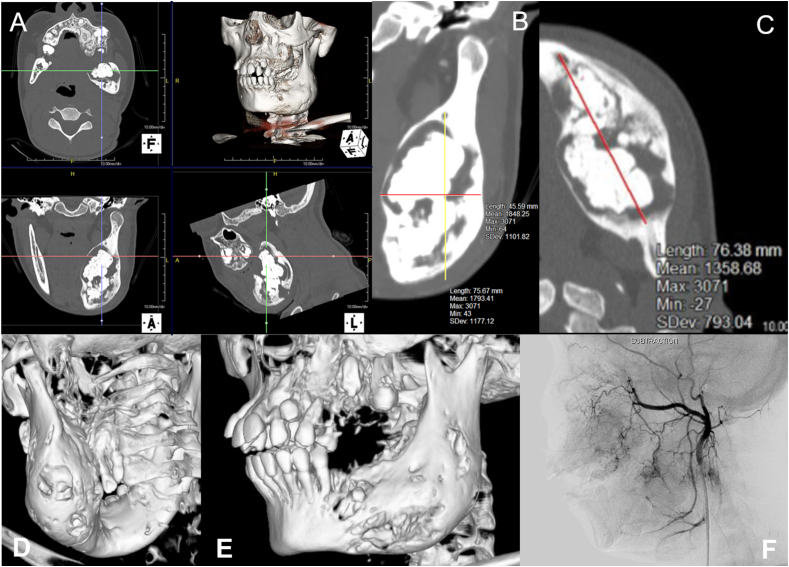
Preoperative findings A,B,C: Preoperative computed tomography image showing the size of the mandibular tumor. D,E: Preoperative 3-dimensional computed tomography image showing significant enlargement of the mandibular tumor. F: Angiographic computed tomography image at the time of embolization, showing dilated peripheral blood vessels and abundant blood flow into the lesion.

**Fig. 5:**
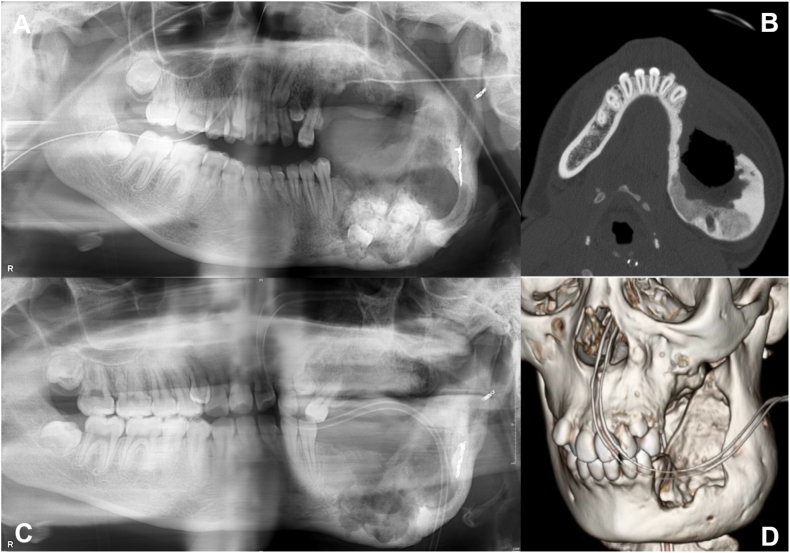
Postoperative findings. A: Panoramic radiography showing 2/3 of the odontoma was removed after the 1st surgery. B: Horizontal computed tomography image showing good bone formation in the mandible 3 months after the 1st surgery. C,D: Panoramic radiography and 3-dimensional computed tomography after 2nd tumor removal surgery showing complete removal of the mandibular odontoma.

**Fig. 6:**
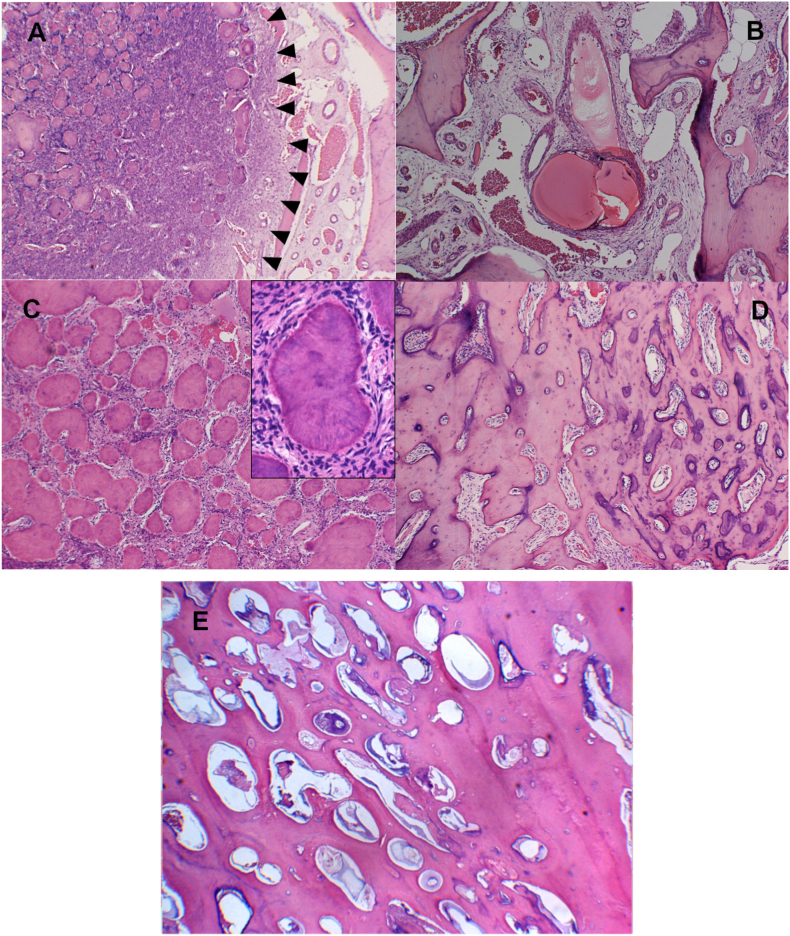
Cemento-ossifying fibroma of the maxilla and odontoma of the mandible. A: Dense fibroblastic cell proliferation with numerous cementum-like calcified materials is observed(H&E; original magnification × 20). The demarcation between the tumor and normal bone is generally clear (A, *arrowheads*). B: In the surrounding normal bone, dilatated abnormal veins and capillaries are seen (H&E; × 200). C: Numerous cementum-like calcified materials are distributed (H&E; × 100). It is surrounded by an uncalcified rim and cementoblastic cells (C, inset, original magnification × 200).D: Bone-like trabeculae are also observed (H&E; × 100). E: The tumor consists of irregular enamel, dentin, and odontogenic epithelium (H&E; × 100).

**Fig. 7:**
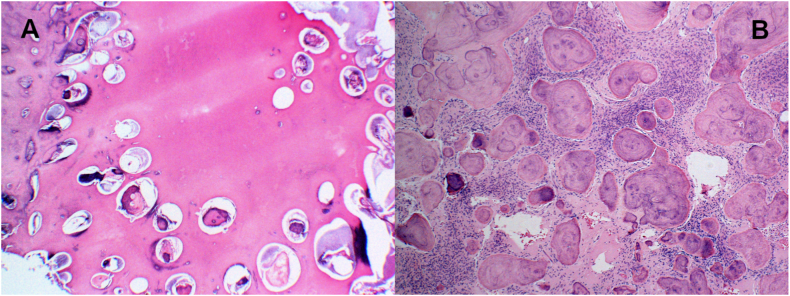
A: COF in maxilla contains odontoma component (H&E; x40). B: Odontoma in mandible contains COF component (H&E; x100).

The mandibular tumor had an enlarged peri-transmission zone around the hard tissue in the preoperative CT images, and the lesion was approximately 7.6 × 7.5 × 4.5 cm in size (Fig. 4AB and C). 3D constructed images also showed a markedly enlarged mandibular tumor (Fig. 4 D and E). Four days before surgery, the left temporomandibular and inferior alveolar arteries were super selectively embolized in the neurosurgery department. Angiographic CT findings at the time of embolization showed dilated peripheral blood vessels and abundant blood flow, consistent with the areas corresponding to the left maxillary and mandibular lesions (Fig. 4F). Subsequently, left-sided maxillary and mandibular tumor resections and tracheotomies were performed (Fig. 5A). At the time of the first biopsy, the maxillary tissue was soft, but it became harder on each successive biopsy and excision. Three months postoperatively, good bone formation was observed in the area where the mandibular odontoma was removed (Fig. 5B). Therefore, the remaining odontoma was removed, and the tracheotomy was closed (Fig. 5C and D). Histopathologically, the maxillary tumor was generally well-demarcated from the surrounding normal bone (Fig. 6A). Numerous cementum-like calcified materials were observed within fibroblastic cell proliferation (Fig. 6A and C). Dilated abnormal veins and capillaries were observed at the boundary area with the normal bone (Fig. 6B). Cementum-like calcified materials (Fig. 6C) and massive bone-like trabecular structures (Fig. 6D) were observed in the central portion of the tumor. The cementum-like calcified material was surrounded by uncalcified rims and cementoblastic cells (Fig. 6C (inset)). The final diagnosis of the maxillary tumor was COF. The mandibular tumor was diagnosed as a complex odontoma (Fig. 6E). Interestingly, detailed observations showed small foci of odontoma in the maxillary COF (Fig. 7A) and small COF-like areas in the mandibular odontoma (Fig. 7B). Thereafter, the infection was controlled, and the patient showed good bone formation 1 year and 8 months postoperatively. The maxilla also showed no increase in lesions, and both the upper and lower jaws have a good prognosis.

## 3 Discussion

### 3.1 Maxillary lesion: cemento-ossifying fibroma (COF)

At the time of biopsy, a juvenile psammomatoid ossifying fibroma (JPOF, WHO 2017) was suggested as a differential diagnosis due to a large lesion extending over the entire left maxilla in a young patient and pathological findings (Fig. 3A and B). However, this ultimately resulted in a COF. JPOF is a clinicopathological variant of ossifying fibroma and bone-related lesions [10]. It is recognized that each tumor has distinct clinical and histopathological characteristics.

JPOF usually develops at an average age of 16–33 years and rapidly increases over a short period [11]. However, in the present case, it may have occurred between the ages of 7 and 11 years in the tooth-bearing area of the premolars on the left side of the maxilla. The tumor pushed the impacted left maxillary second premolar upward. The rapid expansion of the lesion may have been due to increased blood flow during the mixed dentition period. JPOF predominantly affects the paranasal sinuses and occurs in the maxilla, mandible, orbit, and frontal bones. There have been no reports of malignant transformation; however, the recurrence rate is high (approximately 30%–58 %). Since local invasiveness is observed, complete removal is recommended; however, curettage, excision, or partial resection may also be selected depending on the site and the individual patient's condition [12,13]. In contrast, COF, which was the final diagnosis, is a benign odontogenic mesenchymal tumor arising in the tooth-bearing area of the jawbone. COF consists of bone- or cementum-like hard tissue formed in proliferating fibroblasts originating from the periodontal ligament. It is rare, and the predilection for lesions occurs in the third and fourth decades and is more common in women. The incidence is higher in the mandible than in the maxilla [10]. Histopathologically, the tumor was generally well demarcated from the surrounding alveolar bone (Fig. 6A), and numerous vascular malformations were identified at the border between the tumor and normal jawbone (Fig. 6B). The tumor was composed of a dense proliferation of neoplastic fibroblast-like cells and various amounts of cementum-like and bone-like hard tissues, with rimming of the cementoblasts and osteoblasts (Fig. 6A–D). Moreover, consistent with the progressive clinical sclerosis of the tumor, bone- and cementum-like aggregate structures were also observed in the center of the lesion (Fig. 6C). The clinical behavior of the tumor and radiographic and histological findings of the surgical specimen differed from those of JPOF during the follow-up period from biopsy to excisional surgery. Here, we discuss the clinical, radiographic, and histological findings. Diagnosis based on histological findings alone is difficult because of similar findings in jawbone lesions. A comprehensive diagnosis must be made, including clinical and imaging findings. The final pathological diagnosis was COF.

The postoperative healing was excellent, and no deep extension of the lesion has been observed to date. However, there is no dispute that COF requires close and long-term follow-up, as malignant transformation has been reported [14]. In cases where a definitive diagnosis is difficult, genetic testing should be added. Mutations in CDC73 have been reported in COF, but no gene search was performed at this time.

In summary, an accurate diagnosis of COF and JPOF can be made using not only biopsy, but also a comprehensive evaluation of imaging findings and clinical characteristics. COF requires strict long-term follow-up because of the possibility of malignant transformation. In addition, treatment options should be selected on a case-by-case basis, considering the location and extent of the lesions. In this case, the appropriate surgery was selected based on imaging findings, biopsy results, and symptom progression, with favorable results.

### 3.2 Mandibular lesion: giant odontoma

Odontomas are considered tissue malformations (malformations of the tooth) consisting of hard dental tissue, including enamel, dentin, cementum, residual odontogenic epithelium, and ectomesenchyme. Based on their characteristics, they can be classified into compounds and complexes. There was no sex difference in the occurrence, and the majority were detected by the age of 20 years. The most common site of development was the maxillary anterior in the compound type, whereas the mandibular molars and maxillary anterior were more common in the complex type [10]. Most tumors are less than 3 cm in size, and giant odontomas larger than 3 cm are rare [15,16].

Studies reporting giant mandibular odontomas larger than 3 cm since 2010 are summarized in Table 1. The largest sizes reported were 8 × 6 × 4 cm [17], 8 × 4 cm [18], and 8 cm [19]. In the present case, the size was measured on preoperative CT images and was 7.6 × 7.5 × 4.5 cm (Fig. 4B and C). The youngest reported case of giant odontoma was in a 7-year-old child [18], but in this case, the lesion was already identified as a giant odontoma at the age of 6 years. Regarding surgical procedures, unilateral hemimandibulectomy was performed in only one case; otherwise, tumor removal procedures were carried out in all cases to maintain mandibular continuity [17]. In two cases, the two-stage tumor removal technique was chosen, as in the present case, mainly to avoid fractures [20,21]. The first report of two-stage tumor resection for a giant odontoma was published in 2005 [22]. Because odontomas are benign tumors and are detected and treated at a relatively young age, there are many advantages to choosing a technique that does not involve segmental mandibulectomy and reconstruction and preserves mandibular continuity whenever possible. Histopathologically, the lesions predominantly contained complex odontomas composed of irregular enamel, dentin, and cementum structures (Fig. 6E).

**Table 1:** Papers reported as giant odontoma≧3cm in size since 2010.

	Study, Year published(Location)	Gender	Age	Size(cm)	Site of lesion	Type	Surgery
１	Soliman et al., 2022(Syria) [23]	Male	8	4.4	Right side molar	Complex	Tumor removal, extraoral approach
２	Samieirad et al., 2022(Iran) [24]	Female	16	4.5 × 4	Right side	Complex	Tumor removal intraoral approach + iliac bone graft
３	Balaji and Balaji 2021(India) [25]	Female	24	3.3 × 2.2 × 2.1	Right side molar	Complex	Tumor removal, intraoral approach + rib graft
４	Aschaitrakool et al., 2021(Thailand) [26]	Female	19	4 × 3	Right side molar	Complex	Tumor removal, extraoral approach + plate reconstruction
５	Bueno et al., 2020(Brazil) [19]	Male	42	8	Left side of mandibular angle	Complex	Tumor removal, extraoral approach
６	Botelho et al., 2019(Portugal) [27]	Female	53	3.1 × 2.3	Right side molar	Complex	Tumor removal intraoral approach
７	Saravanan et al., 2019(India) [20]	Female	12	4 × 3	Left side of mandibular angle	Complex	Tumor removal, intraoral approach (2 stage surgery)
８	Park et al., 2018(Korea) [15]	Female	28	3 × 2.5 × 2	Right side molar	Complex	Tumor removal, intraoral approach
９	Akerzoul et al., 2017(Morocco) [28]	Male	35	6 × 6	Right side molar	Complex	Tumor removal, intraoral approach
10	Widayanti et al., 2017(Indonesia) [17]	Female	17	8 × 6 × 4	Left side	Complex	Mandibular hemicolectomy + plate reconstruction
11	Singh et al., 2016(India) [29]	Female	18	6 × 3	Left side	Complex	Marginal resection, extraoral approach + iliac bone graft
12	Bagewadi et al., 2015(India) [30]	Male	22	4	Right side molar	Complex	Tumor removal, intraoral approach
13	Perumal et al., 2013(South Africa) [31]	Female	24	5.5 × 4 × 2.5	Right side molar	Complex	Tumor removal, intraoral approach
14	D'Cruz et al., 2013(India) [32]	Male	18	4.5 × 3 × 3	Left side of mandibular angle	Complex	Tumor removal, intraoral approach
15	Lehman et al., 2013(Israel) [18]	Female	７	8 × 4	Right side molar	Compound	Tumor removal, intraoral approach
16	Spini et al., 2012(Brazil) [16]	Male	９	6	Anterior region	Complex	Tumor removal, intraoral approach
17	Chrcanovic et al., 2010(Brazil) [21]	Male	21	4 × 2.8 × 3	Right side of mandibular angle	Complex	Tumor removal, intraoral approach (2 stage surgery)
18	Biocic et al., 2010(Croatia) [33]	Male	10	5 × 3	Right side	Complex	Tumor removal, intraoral approach
	Our case	Female	14	7.6 × 7.5 × 4.5	Left side	Complex	Tumor removal, intraoral approach (2 stage surgery)

Based on these findings, the most common treatment method for giant odontoma in the mandible is to remove the tumor while preserving mandibular bone continuity. The two-stage tumor removal technique, which was also performed in this case, is a beneficial technique that avoids fracture and reconstruction.

### 3.3 Association of Sturge-Weber syndrome (SWS) with maxillary and mandibular bone lesions

To date, no published papers show an association between SWS and COF. However, there is a paper suggesting an association between the syndrome and juvenile ossifying fibroma (JOF) in areas consistent with port wine spots on the face [34]. Neurocutaneous syndrome, which also includes SWS, has been reported to be associated with ossifying fibroma (COF in the current WHO classification), which occurs in tooth-bearing areas, as in the present case [35], and odontoma [36]. In the present case, there were no port wine spots, which are characteristic of SWS; however, according to the Roach classification, there were cases in which port wine spots were absent [37]. In the present case, all lesions were located on the left side. Histopathologically, the maxillary COF contained an odontoma, and the mandibular odontoma contained a COF component (Fig. 7A and B), confirming the coexistence of COF and odontoma in both maxillary and mandibular lesions. Furthermore, CT tomography at the time of embolization showed that the peripheral vessels in the lesion were dilated, indicating abundant blood flow (Fig. 3F). Histologically, numerous distorted and greatly dilated vessels were observed at the border between the lesion and surrounding normal bone (Fig. 6B), suggesting that the lesion coincided with a capillary malformation site. Abundant blood flow was observed in areas consistent with maxillary and mandibular lesions, suggesting that these lesions may be associated with SWS, which is primarily a vascular lesion caused by vascular abnormalities. Radiographs at 6 years of age showed that the left lower second deciduous molar was deficient (Fig. 2A). Embryologically, the tooth germs of deciduous teeth are generated by 6 weeks of fetal life. If it does not develop at that time, it will be missing. SWS is thought to be due to failure of venous development associated with failure of retraction of the primary venous plexus, which is believed to occur between 5 and 8 weeks of fetal life [5,6]. Therefore, tooth loss and SWS may be influenced by environmental or genetic factors that occur simultaneously. Collectively, it is possible that the same side of the maxillary COF and mandibular odontoma in the present case were not separate lesions, but rather similar lesions with a common genetic abnormality arising in odontogenic mesenchymal cells. Recently, the association of the GNAQ gene with port wine spots, which are capillary malformations of the skin and intracranial chondromalangiomas, has been clarified [38]. A limitation of this case report is that genetic abnormalities were not investigated; however, we hope to have the opportunity to further analyze the relationship between the lesions and the syndrome, including testing for the aforementioned genes.

## 4 Conclusion

In this case, we concluded that the maxillary and mandibular bone lesions are a series of lesions associated with SWS. In addition, in future research and diagnosis, it is important to observe blood flow abnormalities in detail and understand how they relate to SWS and jawbone lesions. The accumulation of such case reports can provide clinicians with references and treatment options for similar cases. This will enable the development of more effective treatments and new strategies to prevent lesion progression.

## Informed consent

Proper informed consent for this case report was obtained from the patient and patient's mother in writing.

This case was presented as a poster at the 21st International Congress of Oral Pathology and Medicine (August 17–21, 2023, Taipei, Taiwan).

Oral presentations were made at the 67th Congress of the Japanese Society of Oral and Maxillofacial Surgeons (Nov. 4–6, 2022, Chiba, Japan) and at the joint meeting of the 33rd Annual Meeting of the Japanese Society of Oral Pathology, the 35th Annual Meeting of the Japanese Society of Oral Diagnosis, and the 32nd Annual Meeting of the Japanese Society of Oral Medicine (Sept. 23–24, 2022, Sapporo, Japan).

## Written consent for publication

Written informed consent for the publication of all data and images in this case report was obtained from the patient and the patient's guardian prior to submission.

## Data availability statement

The data are included in the article/supplementary material/referenced in this article. The data associated with this case report have not been deposited in a publicly available repository because of the nature of this study.

## CRediT authorship contribution statement

**Aki Sato:** Writing – original draft, Visualization, Funding acquisition. **Hisako Furusho:** Writing – original draft, Visualization. **Tatsushi Matsumura:** Writing – review & editing, Supervision. **Makoto Nakano:** Writing – review & editing. **Koichi Sawaki:** Writing – review & editing. **Yohsuke Yoshioka:** Writing – review & editing. **Sho Akashi:** Writing – review & editing. **Mutsumi Miyauchi:** Writing – review & editing, Supervision. **Nobuyoshi Mizukawa:** Writing – review & editing, Supervision. **Seiji Iida:** Writing – review & editing, Supervision, Project administration.

## Declaration of competing interest

The authors declare that they have no known competing financial interests or personal relationships that could have appeared to influence the work reported in this paper.
